# Population-based overall and net survival of childhood leukemia at 1-, 5-, and 10-years of follow-up in three regions of Colombia

**DOI:** 10.3332/ecancer.2024.1759

**Published:** 2024-09-06

**Authors:** Natalia Godoy-Casasbuenas, Fabian Gil, Nelson Arias, Claudia Uribe Pérez, Harold Mauricio Casas Cruz, Luisa Bravo Goyes, Esther de Vries

**Affiliations:** 1 Ph.D. Program in Clinical Epidemiology, Department of Clinical Epidemiology and Biostatistics, Faculty of Medicine, Pontificia Universidad Javeriana, Bogotá, Colombia; 2 Department of Clinical Epidemiology and Biostatistics, Pontificia Universidad Javeriana, Bogotá, Colombia; 3 Population-Based Cancer Registry of Manizales, Health Promotion and Disease Prevention Research Group (GIPSPE). Instituto de Investigaciones en Salud, Departamento de Salud Pública, Universidad de Caldas, Manizales-Colombia; 4 Population-Based Cancer Registry of the Metropolitan Area of Bucaramanga, Universidad Autónoma de Bucaramanga, Bucaramanga, Colombia; 5 Population-Based Cancer Registry of Pasto, Centro de Estudios en Salud (CESUN), Grupo de investigación salud pública, Universidad de Nariño, Pasto, Colombia

**Keywords:** relative survival, overall survival, childhood leukemia, population-based cancer registry

## Abstract

**Background:**

Childhood leukemia (CL) is the most common type of childhood cancer worldwide and in Colombia. Thanks to therapeutic innovations and improved access, the survival of children and adolescents with leukemia has increased considerably worldwide, especially in high-income countries. In Colombia, a middle-income country, survival has also been observed to increase in big cities. However, the survival rate in intermediate cities is still unknown.

**Objective:**

This study aimed to assess short- and long-term survival rates of children with leukemia coming from three intermediate Colombian cities as well as to compare overall survival (OS) rates versus relative survival (RS) rates of this population of children.

**Methods:**

Data from population-based cancer registries in three Colombian cities (Bucaramanga metropolitan area, Manizales and Pasto) were analyzed. OS and RS of up to 10 years were estimated for children who were diagnosed with leukemia at ages 0–18 years between 1998 and 2018 and followed up for vital status. RS was calculated using the Pohar-Perme method. We performed a separate survival analysis by gender and by period of diagnosis (before and after 2010).

**Results:**

We included data from 507 children and adolescents diagnosed with leukemia. RS at 1, 5 and 10 years after diagnosis were similar between the populations for the respective timeframes (RS Bucaramanga 86.5%, 66.9% and 52.5%; Manizales 81.1%, 62.8% and 61.1%; Pasto 81.7% at 1 year, 66.2% at 5 years and 59.4% at 10 years). OS and RS were very similar for all estimates and periods. There were no clear differences in RS between genders across the three population-based cancer registries and there was an improvement in RS after 2010, particularly in Bucaramanga and Pasto.

**Conclusion:**

Our study reports similar 5-year survival rates for CL in these Colombian cities compared to rates documented in other Latin American countries and larger Colombian cities. These are far below what is reported in high-income settings. This highlights opportunities for improvement in the Colombian health system, where numerous barriers persist in terms of suspicion, diagnosis and continuity of treatment for CL.

## Introduction

The treatment of childhood cancer is one of the great success stories of modern oncology [1, 2]. Before the 1970s, most children or young adults diagnosed with cancer had little chance of being cured [3]. Since then, 5-year relative survival (RS) has increased dramatically up to 80% in high-income countries, especially for some types of childhood cancer such as leukemias [4–6]. Indeed, significant improvements in childhood leukemia (CL) survival have been observed over recent decades, primarily attributed to progressive enhancements in multiagent chemotherapy regimens. These improvements have been complemented by the stratification in treatment intensity according to individual patient clinical profiles, biological characteristics of leukemia cells and early treatment response [7, 8]. These factors collectively serve as predictive indicators for the risk of relapse, shaping more effective therapeutic strategies. In high-income settings, 5-year CL survival rates have reached levels of 80%–90% [9]. However, these survival rates are significantly poorer and varied in lower- or middle-income settings.

In Latin America, the 5-year net survival rates for acute lymphoblastic leukemias (all ages) varied from 49.8% in Ecuador to 93.1% in Puerto Rico for children diagnosed during 2010–2014 [10, 11]. There has been a progressive improvement in CL survival rates in Colombia. Population-based data have shown that there was an improvement in the 5-year net survival for acute lymphoid leukemia (ALL) diagnosed in children up to 14 years of age within the country rising from 52.3% in the period between 2000 and 2004 to 68.9% between 2010 and 2014 [10]. This improvement in survival has been due to the universalization of the Colombian health system, improved access to diagnostic technology, greater availability of drugs (allowing the implementation of multimodal and more intensive regimens), better supportive care and blood banking and also an increase in trained human resources [12–14]. Indeed, to achieve and maintain remission, all patients diagnosed with ALL in Colombia must adhere to a chemotherapy protocol. This protocol typically includes the four main elements established by most international cooperative research groups: induction, post-induction (which includes consolidation and reinduction), central nervous system prophylaxis and maintenance therapy [15]. In addition, the Colombian health system has implemented a range of public health normative laws, such as the 2010 Childhood Cancer Law that seeks to reduce the cancer mortality rate in children and adolescents through guaranteed care. Subsequent regulations, like *Resolution 418 of 2014* established a comprehensive care pathway to ensure timely treatment for children with suspected or diagnosed leukemia. Furthermore, the *Statutory Law of 2015,* which regulates the fundamental right to healthcare, aims to reduce the time between suspicion and diagnosis and minimize treatment discontinuation or abandonment for various types of childhood cancer, including leukemia [5, 16, 17].

While advances in CL treatment have significantly improved survival rates, especially in large cities, disparities persist within regions of Colombia. Ramirez *et al* [12, 14] conducted a population-based study to describe childhood cancer survival disparities within a universal healthcare system in Cali, the third-largest city in Colombia. They found significant disparities in survival primarily by insurance and type of tumor which could be partially explained by treatment suspension by some healthcare providers, higher treatment-related mortality and advanced disease at diagnosis [14]. These findings highlight the importance of evaluating survival rates across diverse geographic areas – particularly taking into account that additional geographic and logistical challenges to reach a timely diagnosis and opportune treatment may exist in smaller cities in the country [18]. While the improvements in survival rates in capital cities have been notable [4, 14, 19], the survival rates in intermediate cities such as Bucaramanga, Manizales and Pasto remain unknown.

This study aims to address this gap by, first, assessing the population-based survival of children with leukemia coming from these intermediate Colombian cities. Second, we compared overall survival (OS) rates with RS rates, in order to see if other causes of childhood or adolescent death may partly explain the survival estimates.

## Methods

### Data source

We used data from three Colombian population-based cancer registries of the following cities: Metropolitan area of Bucaramanga (approx. 1,2 million inhabitants: consisting of the municipalities of Bucaramanga, Floridablanca, Girón and Piedecuesta), Manizales (approx. 453,000 inhabitants) and Pasto (approx. 392,000 inhabitants) [20]. These three Population-Based cancer registries (PBCRs) have published their data in Cancer Incidence in five continents Volumes X–XII. They have also participated in the CONCORD-2 and 3 study, the global surveillance of cancer survival from 1995 to 2009 and 2000 to 2014 [10, 21]. For this study, we used the individual-level data coming directly from each PBCR.

CL cases were grouped according to the International Classification of Diseases for Oncology 3rd Edition [22] ranging from 9800-3 to 9948-3: Acute leukemia (9801-3), Lymphoid leukemia (9820-3), Myeloid leukemia (9861-3), Acute myeloid leukemia (9861-3), Acute promyelocytic leukemia (9866-3) and Chronic lymphocytic leukemia (9823-3), among others.

The start of follow-up was determined by the date of diagnosis. For individuals with confirmed histology, this date corresponded to the date of issuing of the pathology report. For those cases lacking histologic confirmation, it was based on the date of clinical diagnosis. The end of follow-up was the date of death (all-cause), the last date of contact or the date of study ending for those alive at that moment. In cases where individuals relocated within the country, updated vital status was available. However, if a cancer patient moved out of the country, their vital status was only confirmed if their death was reported to Colombian authorities. There were 24 cases for which the exact day of diagnosis was not available, but the month of diagnosis was. In these cases, the precise date was assigned as the 15th of the month of diagnosis.

### Statistical analysis

**Figure 1.**
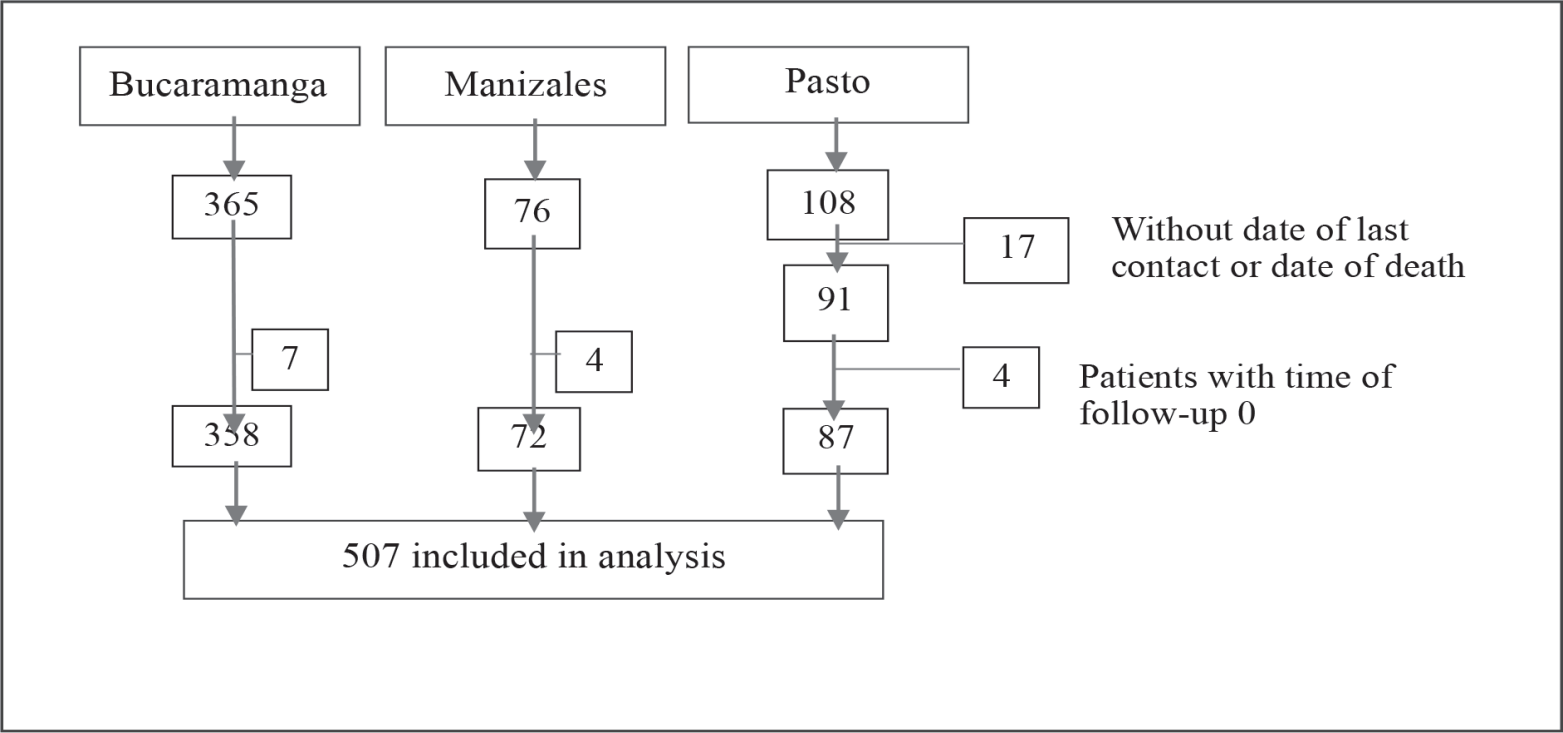
Patient selection flow diagram.

We estimated OS and RS of up to 10 years for children and adolescents (0–18 years) diagnosed during three different time periods: between 2000 and 2017 for Bucaramanga, 2003–2017 for Manizales, and from 1998 to 2018 for Pasto. OS was calculated from the date of diagnosis to the date of death or date of last contact. Patients with neither of both dates were excluded from the analysis (*N* = 17). Patients who died before entering the study (who had a time of follow-up of 0, because their diagnostic test results arrived after their date of death) were also excluded from the analysis (*N* = 15) (Figure 1).

OS (or *crude*) corresponds to the proportion of people diagnosed at age *t* who survive for *d* years after diagnosis. It can also be considered as the probability that a patient who is diagnosed at age *t* is still alive after *d* years [23]. Because it uses the time between diagnosis and date of death from any cause as the endpoint, it is not specific enough to provide information on survival associated with a cancer diagnosis, as a cancer patient may die of cancer or other causes. Cause-specific survival could provide more disease-specific survival information, but considering that the specific cause of death reported on death certificates may suffer from several potential problems regarding the reliability of the exact cause [24] and the availability of detailed information [25], and its calculation implies use of competing risk models, the concept of RS is preferred. RS refers to the ratio of the OS for cancer patients to the expected survival of a comparable group of cancer-free individuals [26]. It provides a measure of excess mortality experienced by cancer patients without requiring cause of death information.

For this study, OS was calculated using the Kaplan-Meier method and we used the Pohar-Perme method for the RS estimation [27]. This non-parametric method operates under the assumption that the time from death due to the disease and death due to other causes are conditionally independent, given a defined set of co-variates. Complete life tables by sex and for single years for the different time periods for each PBCR were employed to calculate the populations´ expected mortality: Bucaramanga: period 2000–2017, Manizales: period 2003–2017, and Pasto: period 1998–2018. Life tables for each region were obtained from the Colombian National Administrative Department of Statistics [28].

We performed a separate survival analysis by gender and by period of diagnosis before and after 2010 which corresponds to approximately half of the follow-up time for most PBCRs to see if there was an observable improvement in survival over time and also more or less coincides with the period prior to the childhood cancer public health normative laws versus the period in which childhood got attention and such norms were gradually formulated and implemented.

Data analysis was performed using strs command [29] in Stata version 17.0 [30]. The age-standardized incidence rate was calculated using the WHO standard population [31]

## Results

### Population characteristics

The average standardized incidence rate per 100,000 person-years (ASIR) of CL was 7.27 during the 2000–2017 period in Bucaramanga, 3.89 in Manizales from 2003 to 2017, and 4.06 during the 1998–2018 period in Pasto (Table 1). Our initial population included 549 cases coming from the three PBCRs. Seventeen patients coming from Pasto were excluded because neither the date of death nor the date of last contact was available. Additionally, 4 patients from Pasto, 4 from Manizales and 7 from Bucaramanga were excluded because they had a follow-up time of 0 (died before entering the study). A total of 507 CL cases were included in the analysis (Figure 1).

The median age at diagnosis was 6 years (Interquartile range (IQR) 3,13) in Bucaramanga, 8 years (IQR: 4, 14) in Manizales and 5 years (IQR: 2,13) in Pasto. In all registries, the majority of patients were males (56%–60%). Over the entire follow-up period, 146 CL patients (40%) from Bucaramanga, 25 (35%) from Manizales and 33 (38%) from Pasto died (Table 1).

### CL survival

The 1, 5 and 10-year OS estimated for children and adolescents diagnosed with leukemia represented in this study were as follows: Bucaramanga: 86.4%, 66.7% and 52.02% – Manizales: 81.0%, 62.9% and 55.4% – Pasto: 81.6, 65.9% and 58.9% (Table 2).

**Figure 2.**
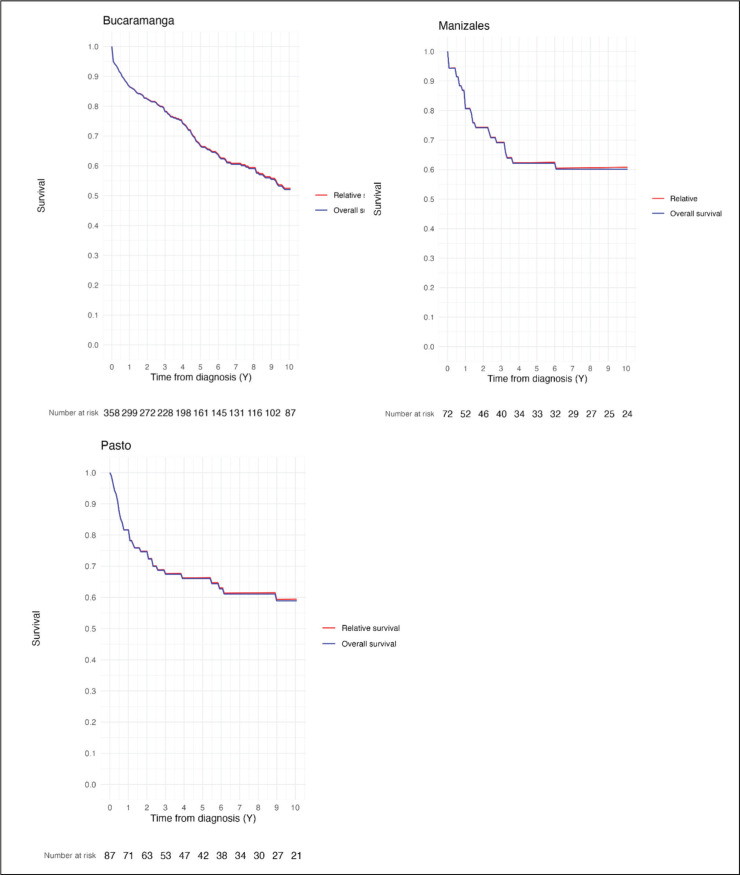
Comparison between OS and RS CL estimates in Bucaramanga, Manizales and Pasto (Colombia).

The 1, 5 and 10-year RS estimated for children and adolescents diagnosed with leukemia represented in this study were as follows: Bucaramanga: 86.5%, 66.9% and 52.5% – Manizales: 81.1%, 62.8% and 61.1% – Pasto: 81.7, 66.2% and 59.4% (Table 2). The similarities between OS and RS estimates are shown in Figure 2.

### CL survival by gender and by diagnostic period

Among the population of children and adolescents diagnosed with leukemia in Bucaramanga, 215 (60%) were males, of which 84 (39%) died, and 143 (40%) were females of which 56 (39%) died. The 1, 5 and 10-year RS among this group was 86.0%, 68.3% and 52.4%. A similar pattern was observed among females, with corresponding RS rates of 87.1%, 65.0% and 52.0%. In Manizales, 42 (55%) of CL patients were male and 34 (45%) were female, with approximately one-third experiencing mortality. RS rates for males at 1, 5 and 10-year were 82.1%, 61.5% and 62.0%, while for females, the rates were 79.8%, 64.0% and 59.6%, respectively. In Pasto, 50 (57%) were male and 37 (43%) were female, with RS rates for males at 1, 5 and 10-year reaching 86.1%, 69.8% and 64.2%, and for females, the rates were 75.7%, 61.6% and 53.3%, respectively.

Concerning CL survival by diagnostic period, more than half of CL cases were diagnosed before 2010: 206 (58%) in Bucaramanga, 40 (53%) in Manizales and 50 (57%) in Pasto.

Notably, in Pasto there was an improvement in RS rates, progressing from 76% to 89% at the 1-year follow-up, followed by increases from 62% to 72% at 5 years and from 58% to 65% at 10 years. In Bucaramanga, RS showed improvement, but this trend was primarily notable during the first year. Conversely, in Manizales, there was a slight decline in RS across the various follow-up periods (Table 3).

## Discussion

In the present study, we aimed to assess the survival rates of children with leukemia coming from three Colombian cities and to compare OS and RS rates. The 5-year net survival rates from the PBCRs of Bucaramanga, Manizales and Pasto were around 60% which is lower than what has been reported in the latest CONCORD study where the survival rates from this disease was around 80% in other Latin American countries like Costa Rica, 76% in Argentina and above 90% in Puerto Rico in the 2010–2014 period. Our findings are similar to the reported net 5-year survival rates in Brazil and Chile whose survival rates were also around 60% [10]. Our findings are also in line with what has been described in the Colombian city of Cali, where the longest PBCR has been established – since 1962, where the OS rate for CL was 65% from 2009 to 2016 [14], suggesting that despite not being the primary capital cities in the country, children and adolescents in the selected cities appear to have at least the same access as in more large cities in the country such as Cali or Bogotá. Previous studies have proposed that in Latin America, children diagnosed in smaller or remote areas may face challenges in accessing healthcare, leading to poorer outcomes [32–34]. Indeed, one of the factors that is likely to contribute to the lower survival rates in our populations compared with countries with more robust healthcare systems, is the often-long period between suspicion and diagnosis of leukemia in Colombian children. Data from the Colombian High-Cost Account for the years 2015 and 2018 show a median time of 31 and 21 days, respectively, which is high compared to the same median time until diagnosis in high-income countries such as Canada where it was 9 days in 2009 [35]. This extended timeframe is closely linked to the risk category assigned at the time of diagnosis. According to the same source, in 2016, 2018 and 2021, 45.2%, 55% and 64.6% of children diagnosed with leukemia were classified as intermediate or high risk at the time of diagnosis [36–38]. Undoubtedly, such delay and risk classification significantly affect the prognosis of these children. Despite all the normative public health measures implemented since 2010 in the country, children and their families still face significant barriers to healthcare access. Some of these obstacles include high co-payments, travel expenses and the need for medical authorizations which lead to fragmentation and treatment interruptions. This persists despite regulations ordering the elimination of such barriers (personal communication with Dr. Harold Casas [39]).

Our data show very similar survival for male and female patients. This finding diverges notably from the trends reported in existing literature, which often indicates poorer survival outcomes for male leukemia patients compared to females [40–42]. The absence of a gender-related survival difference in our study may be due to the small number of CL cases detected in each PBCR and also the short time duration which may have been insufficient to capture any difference. Nevertheless, it justified the decision to conduct all analyses on the overall group rather than stratifying by gender.

RS was higher for children diagnosed after 2010. This improvement was particularly significant in Pasto at 1, 5 and 10 years, and in Bucaramanga, during the first year. We did not detect any difference in Manizales. This finding suggests that with the implementation of the various childhood cancer public health normative laws – the majority of which have been introduced after 2010 – CL patients’ prognosis may have been improved more in the short term than in the long term. However, the available data from the PBCR do not allow us to disentangle the potential mechanisms behind these observations. Caution must be taken when interpreting the 10-year follow-up estimates, treatment options may have improved over time and methodological considerations may also have influenced the estimates: for cases diagnosed before 2010, nearly the entire group had the opportunity for a 10-year follow-up, while for cases after 2010, many have not yet reached this time and many more patients were censored before reaching the 10-year follow-up period.

An interesting finding from our study is that the OS and the RS were very similar in the three PBCRs for the different follow-up periods. OS estimates the chance of remaining alive sometime after diagnosis, whereas RS estimated the chances of surviving cancer while correcting for possible distortions from competing causes of death [26]. It is sometimes suggested that children in low- and middle-income countries (LMICs) have a higher risk of dying in general, which could influence their OS rates: if they die with a certain frequency from causes unrelated to cancer, this will influence the cancerś OS estimates. However, the fact that both types of survival estimates are very similar in our study suggests that the deaths among children and adolescents with leukemia are predominantly due to leukemia itself rather than other unrelated causes. In other words, the observed survival is hardly influenced by other factors outside this disease. This resonates with the findings of a study carried out in Japan which compared three survival measures: overall, cause-specific and RS with data from cancer patients recorded in the Nagasaki Prefecture Cancer Registry. They found that if there were differences between RS and OS rated, this difference represented the proportion of cancer patients who died from other causes. Indeed, the differences between the different survival measures were small at the beginning of the follow-up period and increased with an increase in the number of years of follow-up. These were small for lung and liver cancers, which had a high proportion of death from cancer, whereas the differences were large for prostate cancer, which had a high proportion of death from other causes [43]. A methodological implication of this finding of very similar OS and RS estimates is that, for Colombian childhood cancer populations, it is not needed to perform the difficult task of establishing detailed life tables for children and adolescents in order to calculate the expected survival by calendar year, age and sex [26] but that OS suffices.

Finally, regarding the ASIR of CL, it is noteworthy that although the rates are closely aligned with the values estimated for Colombia by the IARC, at 4.5 per 100,000 individuals [44], there were variations among regions, with Bucaramanga Metropolitan Area having a rate of 7.27 and Pasto 3.69. Bucaramanga’s high ASIR aligns with Rodriguez-Villamizar *et al*’s [45] study on spatial and temporal clustering of CL in Colombia, which identified Bucaramanga and Cali as significant clusters. Bucaramanga’s PBCR covers four municipalities, including rural zones, and Cali is surrounded by sugar cane plantations [46]. Both areas predominantly engage in agriculture involving pesticide use. Further studies are needed to explore the potential relationship between pesticide exposure and CL incidence. However, it is also possible that children with childhood cancer from municipalities close to, but outside of the Bucaramanga metropolitan area, are registered as being residents, thereby inflating numerators but not denominators. The PBCR dedicates great caution in identifying place of residence but sometimes it is impossible to detect such administrative changes.

This study had several limitations: First, the generalizability of the results may be limited given that we only obtained data from three Colombian PBCRs, together covering about 4% of the Colombian population. The healthcare infrastructure, socioeconomic conditions and other factors specific to these cities may not be representative of the broader Colombian or global context. Also, it is important to acknowledge that the information collected by PBCRs has some limitations. Primarily because it focuses on the disease itself and does not include crucial information on access to, type of and continuity of cancer treatment. Including this information in data collection could provide valuable insights into patients’ overall outcomes but is very challenging in a healthcare system where patients are frequently being moved from one healthcare provider to the other. Additionally, the accuracy and completeness of the results heavily rely on the quality of the data collected by the PBCRs. Hence, issues such as underreporting, misclassification, data entry errors or losses to follow-up could impact the reliability of the findings [47]. However, considering the similarities with reports from other, comparable areas of Latin America and between the regions suggests such issues will not have heavily influenced findings. Finally, there was a follow-up duration discrepancy between the different PBCRs with Pasto having the longest follow-up period (starting from 1998 up to 2018), followed by Bucaramanga and Manizales (starting from 2000 to 2003 up to 2017, respectively).

Strengths of this study include the population-based approach of the study, with data from three PBCRs that published their data in the series of Cancer Incidence in Five Continents [48], giving an indication of the reliability, comparability and comprehensiveness of the cancer registry data, ensuring a robust foundation for analysis [49, 50]. Moreover, population-based similarities in survival estimates give some confidence and increase the relevance of our results. Also, the study benefits from a prolonged observation period, allowing for a comprehensive assessment of survival rates over time. Finally, the comparative analysis of both OS and RS rates adds depth to the study, allowing for an initial exploration of the factors influencing CL outcomes in different intermediate Colombian cities.

## Conclusion

In conclusion, our study reports similar 5-year survival rates for CL in the intermediate and small Colombian cities of Bucaramanga, Manizales and Pasto compared to rates documented in large Colombian cities and other Latin American countries. This suggests that despite not being the primary capital cities in the country, children and adolescents in these selected cities appear to have at least the same diagnostic and therapeutic opportunities as in larger cities in the country. These opportunities are far from optimal, as the large differences in survival with children diagnosed in high-income settings illustrate. These differences are not due to childhood and adolescent deaths due to non-related causes, because OS and RS rates were practically equal. This highlights opportunities for improvement in the Colombian health system, where numerous barriers persist in terms of suspicion, diagnosis and continuity of treatment for CL.

## List of abbreviations

CL, Childhood leukemia; OS, Overall survival; PBCR, Population-based cancer registry; RS, Relative survival.

## Conflicts of interest

The authors have no relevant financial or non-financial interests to disclose.

## Funding

The authors declare that no funds, grants or other support were received during the preparation of this manuscript.

## Ethics

This study involves the analysis of secondary data on incidence coming from PBCRs routinely collected and publicly available at the SICC without sharing patients’ personal information and written informed consent was not necessary.

## Author contributions

NGC, EdV, CJR and FG devised and conceived the study, the main conceptual ideas and design of the study. NA, CUP and LB provided the data from PBCRs. NGC and FG performed the data analysis. NGC drafted the original manuscript. All co-authors read and approved the final manuscript.

## Data availability

The CL incident data used for Manizales, Pasto and Bucaramanga are the property of each PBCR, and restrictions apply to the availability of these data, which were used under license for the current study, and so are not publicly available. Individual registries (coauthors in this study) can evaluate reasonable requests for data one-by-one basis.

## Figures and Tables

**Table 1.** Patient demographics and survival outcomes in CL from PBCRs in Bucaramanga, Manizales and Pasto (Colombia).

	Bucaramanga	Manizales	Pasto
Period	2000–2017	2003–2017	1998–2018
ASIR	7.27	3.89	3.61
Patients included	358	72	87
Gender	M: 215 (60%)	M: 40(56%)	M: 50 (57%)
	F: 143 (40%)	F: 32 (44%)	F: 37 (43%)
Age (median), IQR	6 (3.13)	8 (4.14)	5 (2.13)
Alive	219 (60%)	47 (65%)	54 (62%)
Death	146 (40%)	25 (35%)	33 (38%)
Median follow-up time	4.5	3.6	4.9

Note: ASIR, age standardized incidence rate

**Table 2.** CL in Bucaramanga, Manizales and Pasto (Colombia): OS and RS (%) at 1, 5 and 10 years after diagnosis.

Years of follow-up	Bucaramanga		Manizales		Pasto	
	Overall	Relative	Overall	Relative	Overall	Relative
1	86.38	86.46	81.02	81.13	81.61	81.72
5	66.69	66.94	62.49	62.78	65.94	66.22
10	52.02	52.45	60.48	61.12	58.90	59.42

**Table 3.** Net CL survival by gender and diagnostic period in Bucaramanga, Manizales and Pasto (Colombia).

PBCR		Gender		Year of diagnosis	
		Male *N* (%)	Female *N* (%)	Before 2010 *N* (%)	After 2010 *N* (%)
Bucaramanga					
	Diagnosed	215 (60%)	143 (40%)	206 (58%)	152 (42%)
	Deceased	84 (39%)	56 (39%)	96 (47%)	44 (29%)
	Alive	131 (61%)	87 (61%)	110 (53%)	108 (71%)
	Years of follow-up: 1	86.03	87.1	80.1	95.3
	5	68.3	65.0	67.6	58.2
	10	52.4	52.0	54.3	38.1
Manizales	Diagnosed	42 (55%)	34 (45%)	40 (53%)	36 (47%)
	Deceased	14 (35%)	11(34%)	13 (33%)	15 (42%)
	Alive	26 (65%)	21 (66%)	27 (67%)	21 (58%)
	Years of follow-up:				
	1	82.1	79.8	88.4	74.0
	5	61.5	64.0	61.6	62.3
	10	62.0	59.6	62.0	59.0
Pasto	Diagnosed	50 (57%)	37(43%)	50 (57%)	37 (43%)
	Deceased	17 (34%)	16 (43%)	22 (44%)	11 (30%)
	Alive	33(66%)	21 (57%)	28 (56%)	26 (70%)
	Years of follow-up:				
	1	86.1	75.7	76.2	89.3
	5	69.8	61.6	62.0	72.0
	10	64.2	53.3	55.8	65.0

## References

[1] Oeffinger KC, Nathan PC, Kremer LC (2010). Challenges after curative treatment for childhood cancer and long-term follow up of survivors. Hematol Oncol Clin North Am.

[2] Winther J, Kenborg L, Byrne J (2015). Childhood cancer survivor cohorts in Europe. Acta Oncol.

[3] Robison L, Mertens A, Boice J (2002). Study design and cohort characteristics of the childhood cancer survivor study: a multi-institutional collaborative project. Med Pediatr Oncol.

[4] Bravo L, García L, Collazos P (2013). Epidemiología descriptiva de cáncer infantil en Cali, Colombia 1977–2011. Colomb Méd.

[5] Congreso de Colombia (2010). Ley 1388 del 26 de Mayo de 2010 "Por el derecho a la vida de los niños con cáncer en Colombia".

[6] Pardo C, Cendales R (2015). Primera edición edn (Bogotá: Instituto Nacional de Cancerología) p 148. Incidencia, mortalidad y prevalencia de cáncer en Colombia, 2007–2011.

[7] Hunger SP, Mullighan CG (2015). Acute lymphoblastic leukemia in children. N Engl J Med.

[8] Tai EW, Ward KC, Bonaventure A (2017). Survival among children diagnosed with acute lymphoblastic leukemia in the United States, by race and age, 2001 to 2009: findings from the CONCORD-2 study. Cancer.

[9] Pui CH (2020). Precision medicine in acute lymphoblastic leukemia. Front Med.

[10] Allemani C, Matsuda T, Di Carlo V (2018). Global surveillance of trends in cancer survival 2000–14 (CONCORD-3): analysis of individual records for 37 513 025 patients diagnosed with one of 18 cancers from 322 population-based registries in 71 countries. Lancet.

[11] Guzman CP, Cordoba MA, Godoy N (2020). Childhood cancer in Latin America: from detection to palliative care and survivorship. Cancer Epidemiol.

[12] Ramirez O, Aristizabal P, Zaidi A (2018). Implementing a childhood cancer outcomes surveillance system within a population-based cancer registry. J Global Oncol.

[13] Piñeros Petersen M (2011). El abandono del tratamiento en los niños con cáncer: un reto para todos. Rev Colomb Cancerol.

[14] Ramirez O, Aristizabal P, Zaidi A (2018). Childhood cancer survival disparities in a universalized health system in Cali, Colombia. Pediatr Hematol Oncol J.

[15] Ministerio de Salud y Protección Social de Colombia (2013). Guía de práctica clínica para la detección oportuna, diagnóstico y seguimiento de leucemia linfoide aguda y leucemia mieloide aguda en niños, niñas y adolescentes. Para uso de profesionales de salud.

[16] (2014).

[17] (2015).

[18] de Vries E, Buitrago G, Quitian H (2018). Access to cancer care in Colombia, a middle-income country with universal health coverage. J Cancer Policy.

[19] Sarmiento-Urbina I, Linares-Ballesteros A, Contreras-Acosta A (2019). Resultados del Protocolo ACHOP 2006 en los niños con leucemia linfoblástica aguda en la Fundación HOMI Hospital de la Misericordia de Bogotá, en el periodo 2007–2012. Iatreia.

[20] Bray F, Colombet M, Aitken J (2023). https://ci5.iarc.who.int.

[21] Allemani C, Weir HK, Carreira H (2015). Global surveillance of cancer survival 1995–2009: analysis of individual data for 25,676,887 patients from 279 population-based registries in 67 countries (CONCORD-2). Lancet.

[22] Organization WH (2013). International Classification of Diseases for Oncology (‎ICD-O).

[23] Kleinbaum DG, Klein M (2012). Survival Analysis: a Self-Learning Text.

[24] Harteloh P, Bruin K, Kardaun J (2010). The reliability of cause-of-death coding in The Netherlands. Eur J Epidemiol.

[25] Gil Laverde J (2019). Importancia del acceso de los registros de cáncer de base poblacional a las estadísticas vitales: barreras identificadas en Colombia. Revista Colombiana de Cancerología.

[26] Mariotto AB, Noone AM, Howlader N (2014). Cancer survival: an overview of measures, uses, and interpretation. J Natl Cancer Inst Monogr.

[27] Perme MP, Stare J, Esteve J (2012). On estimation in relative survival. Biometrics.

[28] Departamento Administrativo Nacional de Estadística (DANE) (2022). Estimaciones del cambio demográfico.

[29] Dickman P, Coviello V (2015). Estimating and modeling relative survival. Stata J.

[30] Stata Corp (2021). Stata Statistical Software: Release 17.

[31] Ahmad OB, Boschi Pinto C, Lopez A (2001). Age Standardization of Rates: a New WHO Standard. GPE Discussion Paper Series, EIP/GPE/EBD.

[32] Uribe Parra D, Pulido Martinez DC, De Vries E (2020). Access to diagnostic facilities in children with cancer in Colombia: spotting opportunity and distance from a sample. Cancer Epidemiol.

[33] Garibotti G, Moreno F, Dussel V (2014). Disparities in pediatric leukemia early survival in Argentina: a population-based study. Rev Panam Salud Publica.

[34] Ahamad A (2011). Geographic access to cancer care: a disparity and a solution. Postgrad Med J.

[35] Dang-Tan T, Trottier H, Mery LS (2008). Delays in diagnosis and treatment among children and adolescents with cancer in Canada. Pediatr Blood Cancer.

[36] Fondo Colombiano de Enfermedades de Alto Costo - Cuenta de Alto Costo (CAC) (2022). Situación del cáncer en la población pediátrica atendida den el SGSSS de Colombia 2021.

[37] Fondo Colombiano de Enfermedades de Alto Costo - Cuenta de Alto Costo (CAC) (2019). Situación del Cáncer en la Población Pediátrica Atendida en el SGSSS de Colombia 2018.

[38] Fondo Colombiano de Enfermedades de Alto Costo - Cuenta de Alto Costo (CAC) (2016). Situación del Cáncer en la Población Pediátrica Atendida en el SGSSS de Colombia 2015.

[40] Pui CH, Boyett JM, Relling MV (1999). Sex differences in prognosis for children with acute lymphoblastic leukemia. J Clin Oncol.

[41] Holmes L, Hossain J, Desvignes-Kendrick M (2012). Sex variability in pediatric leukemia survival: large cohort evidence. ISRN Oncol.

[42] Amini M, Sharma R, Jani C (2023). Gender differences in leukemia outcomes based on health care expenditures using estimates from the GLOBOCAN 2020. Arch Public Health.

[43] Utada M, Ohno Y, Shimizu S (2012). Comparison between overall, cause-specific, and relative survival rates based on data from a population-based cancer registry. Asian Pac J Cancer Prev.

[44] Global Cancer Observatory International Agency for Research on Cancer Estimated number of new cases in 2020, Latin America and the Caribbean, Colombia, both sexes, ages 0–19 2020. https://gco.iarc.fr/today/online-analysis-table?v=2020&mode=cancer&mode_population=continents&population=900&populations=904_170&key=asr&sex=0&cancer=39&type=0&statistic=5&prevalence=0&population_group=0&ages_group%5B%5D=0&ages_group%5B%5D=3&group_cancer=1&include_nmsc=0&include_nmsc_other=1#collapse-group-0-1.

[45] Rodriguez-Villamizar LA, Rojas Diaz MP, Acuna Merchan LA (2020). Space-time clustering of childhood leukemia in Colombia: a nationwide study. BMC Cancer.

[46] Cendales R, Pardo C, Uribe C (2012). Calidad de los datos en los registros de cáncer de base de población en Colombia. Biomedica.

[47] Gil F, Miranda-Filho A, Uribe-Perez C (2022). Impact of the management and proportion of lost to follow-up cases on cancer survival estimates for small population-based cancer registries. J Cancer Epidemiol.

[48] Bray F, Ferlay J, Laversanne M (2015). Cancer incidence in five continents: inclusion criteria, highlights from volume X and the global status of cancer registration. Int J Cancer.

[49] Parkin DM, Bray F (2009). Evaluation of data quality in the cancer registry: principles and methods Part II. Completeness. Eur J Cancer.

[50] Bray F, Parkin DM (2009). Evaluation of data quality in the cancer registry: principles and methods. Part I: comparability, validity and timeliness. Eur J Cancer.

